# Clinical impact and predictors of carotid artery in-stent restenosis

**DOI:** 10.1007/s00415-012-6436-3

**Published:** 2012-02-09

**Authors:** Katrin Wasser, Sonja Schnaudigel, Janin Wohlfahrt, Marios-Nikos Psychogios, Peter Schramm, Michael Knauth, Klaus Gröschel

**Affiliations:** 1 Department of Neurology, University of Mainz, Langenbeckstr. 1, 55131 Mainz, Germany; 2 Department of Neurology, University of Göttingen, Robert-Koch-Str. 40, 37075 Göttingen, Germany; 3 Department of Neuroradiology, University of Göttingen, Robert-Koch-Str. 40, 37075 Göttingen, Germany

**Keywords:** Carotid artery stenosis, Stent, Angioplasty, Restenosis, Stroke, Duplex sonography

## Abstract

To assess the incidence and clinical significance as well as predictors of in-stent restenosis (ISR) after carotid artery stenting (CAS) diagnosed with serial duplex sonography investigations. We analyzed 215 CAS procedures that had clinical and serial carotid duplex ultrasound investigations. The incidence of in-stent restenosis (ISR) and periprocedural as well as long-term clinical complications were recorded. The influence of an ISR on clinical complication was analyzed using Kaplan-Meier curves and clinical risk factors for the development of an ISR with multivariate logistic regression. During a median follow-up time of 33.4 months (interquartile range 15.3–53.7) an ISR of ≥70% was detected in 12 (6.1%) of 215 arteries (mean age of 68.1 ± 9.8 years, 71.6% male). The combined stroke and death rate during long-term follow-up was significantly higher in the group with an ISR [odds ratio (OR): 3.59, 95% confidence interval (CI): 1.50–8.59, *p* = 0.004]. After applying multivariate logistic regression analysis contralateral carotid occlusion (OR 10.11, 95% CI 2.06–49.63, *p* = 0.004), carotid endarterectomy (CEA) restenosis (OR 8.87, 95% CI 1.68–46.84, *p* = 0.010) and postprocedural carotid duplex ultrasound with a PSV ≥120 cm/s (OR 6.33, 95% CI 1.27–31.44, *p* = 0.024) were independent predictors of ISR. ISR after CAS during long-term follow-up is associated with a higher proportion of clinical complications. A close follow-up is suggested especially in those patients with the aforementioned independent predictors of an ISR. Against the background of a lacking established treatment of ISR, these findings should be taken into account when offering CAS as a treatment alternative to CEA.

## Introduction

Atherosclerotic stenosis of the carotid artery is known as a major risk factor for ischaemic stroke. Carotid endarterectomy (CEA) in combination with best medical treatment of cerebrovascular risk factors is considered to be the gold standard for primary and secondary stroke prevention in patients with significant carotid artery stenosis. More recently, carotid angioplasty and stenting (CAS) have emerged as a potentially less invasive treatment alternative. However, the results of randomized controlled studies and subsequent meta-analyses comparing CEA with CAS failed to prove a general superiority of CAS [1–3]. Nevertheless, there is growing evidence that a subgroup of patients aged <70 years may benefit from CAS intervention [3, 4]. A current major drawback is that prospective data with respect to the clinical long-term outcome are sparse and controversially discussed [1]. Especially the occurrence of an in-stent restenosis (ISR) could endanger the long-term efficacy and safety of CAS. Considering the fact that to date there is no established treatment strategy for an ISR, this issue will be of high clinical importance particularly if patients <70 years are preferably being treated with CAS in the future. By now, the exact rate and clinical impact of ISR during long-term follow-up is still unclear, which may in part be attributable to different definitions of the duplex criteria of an ISR during follow-up investigations [5, 6].

Therefore, the current study had three major aims: first, to investigate the incidence of ISR as assessed with serial duplex ultrasonography; second, to evaluate the impact of ISR on clinical complications (stroke or death) during long-term follow-up; third, to analyze clinical predictors for ISR in order to identify patients at greatest risk who are expected to benefit from a rigorous follow-up.

## Methods

### Patients

Within a prospectively created single-center CAS database we conducted a retrospective analysis of a total of 198 patients (215 arteries) that had been treated between May 2003 and June 2010. Patients had undergone a CAS intervention because of a high-grade carotid stenosis defined as ≥70% in symptomatic patients and ≥90% in asymptomatic patients according to the European Carotid Surgery Trial (ECST) criteria. A carotid stenosis was considered symptomatic if the patient had experienced a transient or permanent ipsilateral ocular or cerebral ischemic event within the past 6 months. All patients received information about the different treatment modalities (CEA, CAS and best medical treatment) and their respective advantages and disadvantages, in particularly the potential complications and risks. With respect to the CAS procedure, all patients were informed about the investigational nature of CAS and gave their written informed consent. The current study is in accordance with International Conference on Harmonisation/Good Clinical Practice guidelines and was approved by the local ethics committee.

### Data collection

An experienced stroke neurologist documented clinical data potentially responsible for influencing the occurrence of an ISR at every follow-up visit. The etiology of all stenoses was atherosclerotic and was further subdivided into a naïve carotid stenosis or a post-endarterectomy stenosis (CEA restenosis). The following cerebrovascular risk factors were recorded using history or direct measurements: hypertension (blood pressure ≥140/90 mmHg measured on repeated occasions or presence of antihypertensive drugs), hyperlipidemia (fasting serum cholesterol levels ≥200 mg/dl or statin therapy), diabetes mellitus (HbA1c ≥6.5%, fasting blood glucose ≥120 mg/dl or presence of antidiabetic drugs), smoking (current or within the previous year), coronary artery disease (history of angina, myocardial infarction, percutaneous transluminal angioplasty or surgery), peripheral occlusive arterial disease (history of typical clinical presentation, percutaneous transluminal angioplasty or surgery) and the presence of contralateral carotid disease (as assessed with ultrasound and subdivided into a stenosis ≥70% or occlusion).

The periprocedural 30-day complications were recorded and categorized as stroke (any neurological deficit persistent >24 h) or death (death of any cause). Long-term follow-up complications recorded in the current study were ipsilateral (symptoms corresponding to the treated artery) stroke or death from any cause. Furthermore, the date and character of carotid re-interventions (balloon angioplasty alone, CAS or CEA) and their specific complications (any stroke or death within 30 days of the procedure) were registered.

### CAS procedure

CAS was carried out by experienced interventional neuroradiologists and done under anesthesiological stand-by. All interventions were performed via a transfemoral approach. Stent type and the use of filter-based neuroprotection devices were chosen at the discretion of the interventionalist. Only patients scheduled for elective CAS were recorded; patients in unstable clinical conditions or with stroke in evolution were excluded. All patients received orally administered acetylsalicylic acid (100 mg/day) and clopidogrel (75 mg/day) at least 3 days before the procedure. Clopidogrel was continued for a minimum of 6 weeks after CAS, and aspirin was administered indefinitely. After being routinely monitored in our intensive care or stroke unit overnight for at least 1 day, all patients were discharged afterwards to a normal ward or home. A clinical examination and duplex sonography were performed before discharge to obtain the clinical status of the patient and confirm stent patency.

### Doppler and duplex sonography

The diagnosis of a carotid artery stenosis and an ISR in particular was made by carotid duplex ultrasound imaging using a combination of direct and indirect criteria, and the presence and extent of intrastenotic and poststenotic turbulent flow. In detail, as direct criteria for the local degree of stenosis, the peak systolic flow velocities (PSV) within the stenosis and poststenotic internal carotid artery, the end diastolic flow velocity in the stenosis, the internal carotid artery/common carotid artery PSV ratio, and the prestenotic and poststenotic frequency patterns were determined. The residual vessel lumen in the B image and the color-coded residual vessel area were documented whenever possible. The flow characteristics of the supratrochlear artery and the anterior cerebral artery as well as the pulsatility of the ipsilateral common carotid artery were taken into account as indirect criteria for a higher grade stenosis. As one of the main criteria the degree of carotid stenosis at baseline was graded according to angle-corrected maximum intrastenotic peak systolic velocities according to ECST criteria as follows: baseline stenosis ≥70% = PSV ≥200 cm/s, baseline stenosis ≥80% = PSV ≥300 cm/s, baseline stenosis ≥90% = PSV ≥400 cm/s.

As there is a lack of generally valid ultrasound criteria for the definition of an ISR and the current literature supposes different criteria [5, 6], we used locally adopted criteria with a PSV ≥300 cm/s as a key feature representing an ISR of ≥70%.

All examinations were performed according to a standardized protocol in the same vascular laboratory with the same ultrasound equipment (Acuson Sequoia™ 512, Siemens, San José, CA) under the supervision of an experienced, board-certified vascular neurologist (K.G.).

### Follow-up protocol

All patients were summoned for serial duplex sonography follow-up at the hospital’s outpatient clinic at 3, 6 and 12 months after the CAS procedure and every 6 months thereafter. During these routine postinterventional visits, a neurologist experienced in neurovascular diseases examined each patient and recorded the aforementioned clinical complications.

### Statistical analysis

Nominal variables were expressed as count and percentages, continuous values as mean ± standard deviation (SD) and not normally distributed values as median values with the corresponding interquartile range (IQR), respectively. For univariate comparisons of categorical data, two-tailed chi-square statistics with Yates’ correction and univariate Fisher’s exact test were used. The Fisher’s exact test was applied when the predicted contingency table cell values were less than 5. Non-normally distributed variables were compared using a Mann-Whitney *U* test.

Overall survival Kaplan-Meier curves were obtained using periprocedural (≤30 days) stroke and death as well as ipsilateral stroke and death during long-term follow-up (>30 days) as a combined endpoint. Interaction for the occurrence of an ISR was tested using the Mantel-Cox test. In order to estimate a potential effect of a variable on the occurrence of an ISR during follow-up, we used a multiple binominal regression analysis. All variables with a *p* < 0.1 on the univariate level were included into a multiple binominal regression analysis (*p* to enter = 0.05, *p* to leave = 0.1). A two-sided *p* value of less than 0.05 was considered to indicate a statistically significant difference. All statistical analyses were performed with SPSS (version 17, SPSS Inc., Chicago, IL).

## Results

### Patient characteristics

Two hundred nineteen patients (237 arteries) undergoing elective carotid artery stenting between May 2003 and June 2010 were analyzed. The data of 21 patients (22 arteries) had to be excluded because of a missing Duplex follow-up, yielding a total follow-up rate of 90.7%. Complete clinical follow-up data with a median duration of 33.4 months (IQR 15.0–53.7) were available for all the remaining 215 arteries (mean age of 68.1 ± 9.8 years, 71.6% male).

The detailed patient characteristics are given in Table 1. An ISR ≥70% with a PSV ≥300 cm/s was detected in 12/215 (5.6%) arteries in 198 patients after a median of 8.6 months (IQR 3.4–17.3). In 9/12 patients (75%) a retrograde flow of the ipsilateral supratrochlear artery and/or the anterior cerebral artery could be detected as indirect duplex criteria of a high grade ISR. Contrast-enhanced reference imaging was performed in 10/12 cases (83.3%). A higher grade ISR could be confirmed in 9/10 patients (90%, 7 by digital subtraction angiography and 2 by computer tomography angiography).

**Table 1:** Baseline characteristics of the study population

Variable	Data		
	No ISR	ISR ≥70%	*p* value
*N*	203	12	
Age (years)	68.1 ± 9.8	67.8 ± 6.6	0.928
Male sex	147 (72.4%)	7 (58.3%)	0.328
Weight (kg)	79.4 ± 13.4	82.5 ± 6.6	0.661
Height (m)	170.6 ± 7.6	169.5 ± 3.9	0.782
Left side	115 (56.7%)	4 (33.3%)	0.141
Symptomatic carotid stenosis	154 (75.9%)	7 (58.3%)	0.328
Stroke	94 (46.3%)	3 (25.0%)	0.232
Hemispherical TIA	45 (22.2%)	3 (25.0%)	0.733
Amaurosis fugax	8 (3.9%)	1 (8.3%)	0.410
Hypertension	184 (90.6%)	12 (100%)	0.606
Hyperlipidemia	132 (65.0%)	11 (91.7%)	0.065*
Diabetes	61 (30.0%)	2 (16.7%)	0.516
Tobacco use	59 (29.1%)	5 (41.7%)	0.347
Coronary artery disease	60 (29.6%)	4 (33.3%)	0.753
Peripheral occlusive arterial disease	37 (18.2%)	5 (41.7%)	0.061*
CEA restenosis	15 (7.4%)	4 (33.3%)	0.014*^,†^
Contralateral ICA occlusion	23 (11.3%)	5 (41.7%)	0.011*^,†^
Contralateral ICA stenosis ≥70%	50 (24.6%)	7 (58.3%)	0.017*
Stenosis ≥90% before CAS	86 (42.4%)	6 (50.0%)	0.603
PSV ≥120 cm/s after CAS	12 (5.9%)	4 (33.3%)	0.001*^,†^
Any Stroke or death ≤30 days	14 (6.9%)	2 (16.7%)	0.221
Ipsilateral stroke or death >30 days	22 (10.8%)	4 (33.3%)	0.043
Median follow-up time (month, IQR)	33.4 (15.5–53.9)	20.8 (5.9–41.8)	0.218
Re-interventions	0 (0%)	8 (66.7%)	<0.001

* Factors included in multiple regression analysis (*p* < 0.1 univariate analysis)

^†^Factors remained significant after multiple regression analysis

Considering the development of an ISR, the cardiovascular risk factors hyperlipidemia and peripheral artery occlusion disease as well as an intervention because of a CEA restenosis in comparison to a naïve CAS procedure were statistically significantly more frequent in this group on univariate level (91.7 vs. 65.0%, *p* = 0.07; 41.7 vs. 18.2%, *p* = 0.061; 33.3 vs. 7.4%, *p* = 0.014, respectively). Moreover, patients with an ISR during follow-up more often presented with a contralateral occlusion or stenosis of the ICA ≥70% at the time of CAS and a post-interventional flow acceleration with a PSV ≥120 cm/s (41.7 vs. 11.3%, *p* = 0.01; 58.3 vs. 24.6%, *p* = 0.02; 33.3 vs. 5.9%, *p* < 0.001).

### Clinical complications

During the 30-day periprocedural follow-up time, the overall stroke and death rate was 7.4%. Twelve patients (5.6%) suffered from stroke and five patients (2.3%) died after a median of 3 days (IQR 3–14). One patient had discontinued his antithrombotic medication and subsequently died of a major stroke after developing an acute in-stent thrombosis. One patient died of an intracerebral hemorrhage immediately after CAS. Other causes of death were a traumatic subarachnoid hemorrhage after discharge, a hypopharynx carcinoma, and one patient died of unknown cause. The combined periprocedural stroke and death rate was 6.9% in patients without and 16.7% in those with ISR (*p* = 0.22).

**Fig. 1:**
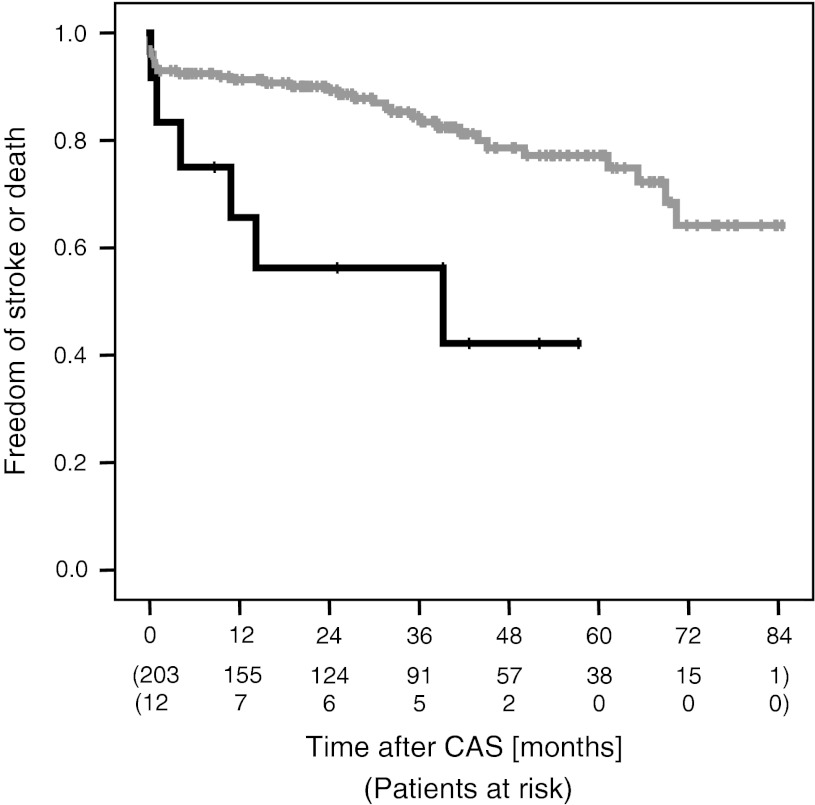
Kaplan-Meier curve representing the freedom of clinical complications (periprocedural any stroke or death and ipsilateral stroke or any death beyond 30 days) patients with (*black*) and without (*grey*) restenosis during follow-up (*p* = 0.004)

During the long-term follow-up period (>30 days after CAS), the combined rate of ipsilateral stroke and death was 10.8% in the subgroup without ISR (7 strokes, 1 stroke followed by subsequent death and 14 deaths) and 33.3% in the group with an ISR ≥70% (2 strokes and 2 deaths; *p* = 0.043) after a median of 31.9 months follow-up time (IQR 16.1–43.3). The cumulative rate of stroke-free survival for patients with and without ISR ≥70% is shown in Fig. 1. Within the patients with ISR there was a statistically significantly higher risk for clinical complications (ipsilateral stroke and death) during follow-up [odds ratio (OR): 3.59, 95% confidence interval (CI): 1.50–8.59, *p* = 0.004]. For the whole study group Kaplan-Meier analysis estimates the freedom of a ≥70% ISR of 96, 95, 94, 93 and 91% after each year.

Within the patients with a restenosis the number of ipsilateral re-interventions during follow-up was significantly higher (0 vs. 66.7%, *p* < 0.001) as compared to those without ISR. In the group with ISR ≥70% an ipsilateral re-intervention was performed in eight patients (3 CAS, 4 PTA, 1 bypass; 66.7%), whereas no interventions of the contralateral side were recorded. Despite the failure of the attempt to recanalize the acute in-stent thrombosis as reported above and the patient’s death 3 days later of a major stroke, there were no other periprocedural complications associated with a re-intervention recorded.

### Carotid stent procedure

A detailed description of the procedure has been described recently [7]. In the vast majority a Carotid Wallstent Monorail^®^ (*n* = 179, Boston Scientific Corp., Natick, MA, USA) was deployed followed by the use of a Zilver^®^ (*n* = 13; Cook Medical Inc., Bloomington, IN, USA) or Precise^®^ stent (*n* = 11, Cordis Endovascular, Warren, NJ, USA). Distal filter-type embolic protection systems were used in 61 of 215 (28.4%) patients. No differences were observed among different stent types, open versus closed cell design or the use of a protection system between the two groups. There was a higher proportion of post-procedural residual stenosis as assessed with duplex sonography (PSV ≥120 cm/s) in the group with subsequent ISR (5.9 vs. 33.3%, *p* < 0.001) compared to those without ISR (see Table 1).

### Independent predictors for ISR

To identify independent predictors for an ISR, a multiple binominal regression analysis was performed including all variables, which were imbalanced (*p* < 0.1 see Table 1) within the univariate analysis (hyperlipidemia, peripheral occlusive artery disease, CEA restenosis, contralateral ICA occlusion/stenosis ≥70%, residual stenosis after CAS as detected by PSV ≥120 cm/s 1–3 days after CAS). The strongest statistically significant predictor for the development of a subsequent ISR after multiple regression analysis was a contralateral carotid occlusion (OR 10.11, 95% CI 2.06–49.63, *p* = 0.004), a CEA restenosis (OR 8.87, 95% CI 1.68–46.84, *p* = 0.010) as an indication for the elective CAS and a postprocedural carotid duplex ultrasound with an elevated PSV ≥120 cm/s indicating a residual low-grade stenosis (OR 6.33, 95% CI 1.27–31.44, *p* = 0.024).

## Discussion

Within the current prospective single-center long-term CAS surveillance we observed significantly more clinical complications (stroke or death) in patients who developed an ISR during follow-up compared to those without ISR. Moreover, a contralateral carotid occlusion, a CAS intervention of a restenosis after CEA and a postprocedural PSV >120 cm/s on duplex sonography indicating a residual low-grade stenosis after CAS could be identified to be independent risk factors for the development of an ISR during follow-up. Against the background of the clinical impact of an ISR, we recommend a tight clinical and ultrasonographic long-term follow-up of patients treated with CAS, especially in those with the aforementioned clinical characteristics.

In the past few years, CAS has frequently been used as an alternative to CEA for the treatment of a carotid artery stenosis yet randomized controlled trials have recently failed to prove a clear benefit in favor for a CAS intervention [1–4]. Especially the long-term benefit of a CAS procedure is currently debated because the CAS data presented to date have reported concerning results [8, 9]. However, according to the current literature a CAS intervention is thought to be effective in younger patients, because two meta-analyses comparing the complications of CAS and CEA showed a trend towards a favorable outcome in patients aged <70 years for those patients treated with CAS [1, 4]. On the other hand, within the recently published long-term results of the SPACE and EVA-3S trials, the incidence of an ISR after 2 years diagnosed with duplex sonography was significantly higher after a CAS intervention compared to CEA (10.7 vs. 4.6%, *p* = 0.009 and 12.5 vs. 5.0%; *p* = 0.02) [8, 10]. In those trials the higher incidence of ISR during follow-up was not found to have an impact on clinical complication rates. Therefore, it has been postulated that restenosis might be a relatively benign pathology [9]. In contrast, our results support the notion that the occurrence of ISR among other factors could endanger the long-term efficacy and safety of CAS, because the long-term risks for stroke or death were significantly higher in patients with an ISR. Against the background that a CAS intervention might be beneficial especially in younger patients, these results should be taken into account for the patient’s individual treatment advice.

One reason for the higher incidence of clinical complications during medium-term follow-up within our single-center experience might be the less well-controlled cardiovascular risk factors after hospital discharge, with, e.g., the lack of routinely scheduled blood samples to adjust current medication such as statins or antidiabetic drugs in our outpatient clinic. The current setting may however reflect common everyday practice in real life and might therefore not necessarily be comparable to the well-structured settings of randomized controlled trials [3, 4, 8]. Moreover, the positive patient selection favoring the compliant and well-educated ones in randomized controlled trials has to be taken into account and could explain the disparity in stroke and ISR rates.

As could be expected, the number of re-interventions (8/12; 66.7%) was higher within the patients with high-grade restenosis in our study. Although no clinical periprocedural complications occurred during routinely scheduled re-interventions within the aforementioned patients, the variety of different treatment strategies selected to engage the ISR (3 re-CAS, 4 PTA and 1 bypass surgery) reflects the fact that an overall accepted treatment strategy for ISR has not yet been established. As within our series, surgical treatment of ISR remained an exception in the reviewed literature because it is technically demanding and can be associated with periprocedural complications [11]. Currently, in most of the cases a PTA or re-CAS is performed [12]. This further highlights the clinical importance of identifying independent risk factors to be able to detect those patients during clinical routine and leading to a thorough long-term sonographic follow-up.

Current data suggest that ISR frequently occurs during the first year of follow-up [5], which is corroborated within the current patient cohort: ISR was observed after a median follow-up of 8.6 months (IQR 3.4–17.3). Not surprisingly, an insufficient result after CAS with elevated PSV >120 cm/s within the stent as detected with duplex ultrasound was found to be associated with subsequent ISR. This is in line with previous studies and may be due to heavily calcified plaques, which made it difficult to establish an appropriate stent positioning without residual narrowing [13, 14]. Interestingly, in a study with 563 patients, Randall et al. found that a residual stenosis of >50% after CAS is associated with an increased risk of ipsilateral stroke in the long run [15]. Therefore, pursuing an optimal stent deployment during CAS seems to be a worthwhile aim, although it is known that an aggressive postdilation bears the risk of distal embolization. It could also result in microvascular injury, which may contribute to an aggravation of inflammatory processes that finally promote neointimal hyperplasia and ISR [7, 16].

Currently, CAS is a recommended treatment alternative for patients with CEA restenosis, because a subsequent CEA bears a higher periprocedural risk than the initial operation [17, 18]. However, in line with our current results, which have identified a previous CEA as an independent risk factor for the development of ISR after CAS, previous authors noted that there may be a higher risk of developing an ISR after stenting of postendarterectomy arteries [18–20]. These findings emphasize that patients with restenosis after CEA and a second CAS intervention are prone to develop a second restenosis.

Another risk factor for ISR identified in this study is the presence of a contralateral carotid artery occlusion. Although higher blood flow velocities in a carotid artery are a well-known phenomenon in case of a contralateral carotid occlusion [21], the restenosis could be confirmed in four of five patients within our patient cohort during conventional angiography (in one patient a confirmation was not possible because of imaging artifacts during contrast-enhanced CT angiography). These imaging results argue against the possibility that an ISR in these patients might be based solely on a false-positive ultrasound measurement triggered by artificially elevated flow accelerations caused by the contralateral carotid occlusion. According to the current literature, some authors state that CAS would be the favorable treatment option in patients with a contralateral carotid artery occlusion [17, 22], because periprocedural complications may be higher during CEA [23]. Nevertheless, the possibility of an elevated flow, which might overestimate a possible ISR in patients with contralateral occlusion, should be kept in mind by clinicians, and even more so the higher incidence of ISR when offering CAS as a treatment option to this patient subgroup.

Despite the strengths of the current study we are well aware of certain limitations: Because of the retrospective analysis of the prospectively recorded data an ascertainment bias cannot be ruled out, although >90% of all patients had been followed up. The lack of generally valid ultrasound criteria for the detection of ISR [5, 6] may have led to different rates of ISR, although we adopted our criteria in accordance to the current literature.

## Conclusions

ISR after CAS occurs frequently within the first year of follow-up and is associated with a higher risk of clinical complications. Considering that a CAS intervention is frequently used as an alternative to CEA especially in patients <70 years, a strict and long-term follow-up is warranted. Especially patients with the presence of a contralateral carotid artery occlusion treated because of a CEA restenosis or with an insufficient postprocedural result (PSV >120 cm/s) are prone to develop an ISR. With respect to the clinical relevance of an ISR and the lack of a commonly accepted treatment strategy, all efforts should be made to carefully follow-up especially these patient subgroups. Duplex sonography with adopted local criteria to identify ISR should be used as a non-invasive, inexpensive follow-up modality after CAS.
